# The Risk of Premature Burial

**Published:** 1914-05-30

**Authors:** 


*To the Editor of* The Hospital.

### The Risk of Premature Burial.

*To the Editor of* The Hospital.

Sir,—I notice that this topic is once again receiving ventilation in your correspondence columns, and with your permission I should like to offer some observations on the matter which seem to me relevant.

To start with, for my own part I quite agree with most of the criticisms of our "haphazard and unscientific" system of death certification and registration; and I think I may say that the great bulk of the medical profession hold the same view. By all means let "Burial Reformer" and everyone else who believes that premature burial is a possibility work for the improvement of the law in this respect.

I do not, however, share the belief of those who belong to the Association for the Prevention of Premature Burial that there is in fact any risk worth considering of that horrible catastrophe in this country. My objection to the present methods of death registration is rather that they do not act as a sufficient check upon crime than that they conduce to the interment of those who are not really dead. I have never come across a case myself, and I have never met any medical man who has done so either, in which there was the very slightest doubt about the fact of death or the very remotest risk of premature burial. Still, if an improved system of registration is ever introduced, by all means let us have such reasonable provisions added as may serve to minimise this risk.

Lastly, I would draw attention to a method of deciding whether or no death has actually occurred which I saw described some months ago in your own columns. It depended on the clotting of the blood in the retinal veins, a phenomenon easily determined by the use of an ophthalmoscope. Whether this test is as infallible as it is claimed to be I do not know, but at least it is worthy of investigation, and of the attention of the burial reformers.—I am, yours faithfully,

Surgeon.

May 22.

[The original reference to the researches to which our correspondent refers is the New York *Medical Record*, May 3, 1913, in which paper an article on coagulation of the blood in the retinal vessels as a sign of death was published by Dr. Morris Kahn. We may point out, however, that the segmented appearance of the retinal vessels after death has been known for a long time; for it is mentioned in Sir William Gower's book on ophthalmoscopy, and we are informed that it was first noticed about fifty years ago. Whether this test of death is infallible is a matter that deserves to be cleared up, and one that should appeal both to burial reformers and to the sceptical correspondent whose letter appears above.—Ed. The Hospital.]

